# Evaluating of BERT-based and Large Language Mod for Suicide Detection, Prevention, and Risk Assessment: A Systematic Review

**DOI:** 10.1007/s10916-024-02134-3

**Published:** 2024-12-30

**Authors:** Inbar Levkovich, Mahmud Omar

**Affiliations:** 1 Tel-Hai Academic College, 2208 Qiryat Shemona, Upper Galilee Israel; 2 Faculty of Medicine, Tel-Aviv University, Tel-Aviv, Israel

**Keywords:** Suicide, Large Language Models, Artificial Intelligence, Systematic Review

## Abstract

Suicide constitutes a public health issue of major concern. Ongoing progress in the field of artificial intelligence, particularly in the domain of large language models, has played a significant role in the detection, risk assessment, and prevention of suicide. The purpose of this review was to explore the use of LLM tools in various aspects of suicide prevention. PubMed, Embase, Web of Science, Scopus, APA PsycNet, Cochrane Library, and IEEE Xplore—for studies published were systematically searched for articles published between January 1, 2018, until April 2024. The 29 reviewed studies utilized LLMs such as GPT, Llama, and BERT. We categorized the studies into three main tasks: detecting suicidal ideation or behaviors, assessing the risk of suicidal ideation, and preventing suicide by predicting attempts. Most of the studies demonstrated that these models are highly efficient, often outperforming mental health professionals in early detection and prediction capabilities. Large language models demonstrate significant potential for identifying and detecting suicidal behaviors and for saving lives. Nevertheless, ethical problems still need to be examined and cooperation with skilled professionals is essential.

**Supplementary Information:**

The online version contains supplementary material available at 10.1007/s10916-024-02134-3.

## Introduction

Suicide is a significant public health issue that accounts for more than 1% of global deaths, with one life lost to suicide every 40 s [1]. Furthermore, suicide is the fourth leading cause of death worldwide among individuals aged 15–29 years [2]. The ability to identify individuals with suicidal thoughts and behaviors is crucial to suicide prevention [3, 4]. Suicidal thoughts involve the consideration of or the desire to end one’s life and range from passive to active ideation [5, 6]. Suicidal behaviors refer to self-directed actions that may be harmful and involve the intention to die [7]. Although the progression from ideation to behavior is not always linear, it is typically perceived as occurring along a continuum ranging from thoughts to attempts or actual deaths by suicide [8]. Several intervention programs have shown great promise in preventing suicide [9, 10]. Ongoing advancements in artificial intelligence, particularly in large language models (LLMs), have played a significant role in the detection, risk assessment, and prevention of suicide [11, 12]. Advanced iterations of these models, such as Generative Pretrained Transformers (GPT), Llama, Bidirectional Encoder Representations from Transformers (BERT), and Claude, utilize their extensive linguistic capabilities to facilitate detection and intervention, which are crucial for saving lives [13, 14]. This review includes both LLMs and other transformer-based language models, such as BERT. While the standard BERT model does not align with conventional definitions of ‘large language models,’ its advanced bidirectional architecture and applications have significantly contributed to advancements in suicide detection and risk assessment, warranting its inclusion among prominent LLMs [15].

Traditional mental health care systems frequently face challenges such as high costs and limited resources, which impede the timely provision of mental health services [16]. LLMs offer an alternative by enhancing accessibility and addressing geographic, financial, and stigma-related barriers, thus enabling personalized early detection and prevention among those at risk for suicide [17, 18]. Nevertheless, research in this field thus far is relatively limited and is subject to cultural and gender biases [19–21]. Moreover, LLMs cannot replace the expertise of psychologists, psychiatrists, and physicians, who provide essential human assistance and rapid diagnosis and are able to integrate clinical knowledge within ongoing therapeutic processes [22, 23].

This review aimed to provide a comprehensive analysis of the role of LLMs in enhancing the understanding, prevention, detection, and treatment of suicide. In recent years, several articles have provided systematic reviews of the use of artificial intelligence in suicide assessment and prevention. For example, Dhelim et al. [24] investigated AI applications in image and voice processing, highlighting promising opportunities for revolutionizing suicide risk assessment [24]. Similarly, Barua et al. [25] surveyed AI tools to detect anxiety and depression, which can lead to suicidal ideation in adolescents [25].

The current systematic review seeks to supplement these articles with a comprehensive review of LLMs tools in order to provide a unique and up-to-date picture of this field.

## Methods

### Registration and Protocol

This systematic review was registered with the International Prospective Register of Systematic Reviews (PROSPERO) under the registration code CRD42024546865 [26]. Our methodology adhered to the Preferred Reporting Items for Systematic Reviews and Meta-Analyses (PRISMA) guidelines [27].

### Search Strategy

We searched seven major databases—PubMed, Embase, Web of Science, Scopus, APA PsycNet, Cochrane Library, and IEEE Xplore—for studies published from January 1, 2018, through April 2024. The primary search terms included ‘large language model,’ ‘LLM,’ ‘GPT,’ ‘ChatGPT,’ ‘Generative Pre-trained Transformer,’ ‘BERT,’ ‘Transformer models,’ ‘RoBERTa,’ ‘AI language model,’ ‘suicide,’ ‘suicidal ideation,’ ‘self-harm,’ ‘suicide attempt,’ and ‘suicidal thoughts.’ The full Boolean search strings for each database are provided in the Supplementary Materials.

This period was selected because it commenced with the initial release of the Generative Pretrained Transformer (GPT) LLM. Our study focused on the impact of integrating LLMs on suicide detection, risk assessment, and prevention. Our search was complemented by manual screening of references and targeted searches using Google Scholar.

### Eligibility Criteria

The search included original research articles and conference papers [28]. The exclusion criteria were limited to preprints, review articles, case reports, commentaries, protocols, editorials, and non-English publications. Initial screening was facilitated using the Rayyan web application [29].

### Selection Process and Data Extraction

Two reviewers (MO and IL) independently conducted the initial screening and study selection according to predefined criteria. Discrepancies were resolved through discussion. The researchers carried out data extraction using a standardized form to ensure consistent and accurate data capture. Extracted data included author names, publication year, sample size, data types, tasks, models used, results, conclusions, and limitations. Discrepancies were addressed through discussions.

### Risk of Bias Assessment

We employed two distinct tools to assess the risk of bias, each tailored to different study designs within our review. The Quality Assessment of Diagnostic Accuracy Studies-2 (QUADAS-2) was applied to diagnostic studies that focused on the detection of suicidal thoughts and behaviors [30]. This tool was specifically used for comparisons between large language models and mental health experts, physicians, or other established reference standards. For studies that were aimed at developing predictive tools using LLMs for the prevention of suicide attempts and that made no direct comparisons with existing methods, we utilized the prediction model Risk Of Bias Assessment Tool (PROBAST) [31]. This multi-tool approach allowed us to appropriately address the diverse methodologies and applications presented in the reviewed studies.

## Results

### Study Selection

**Fig. 1:**
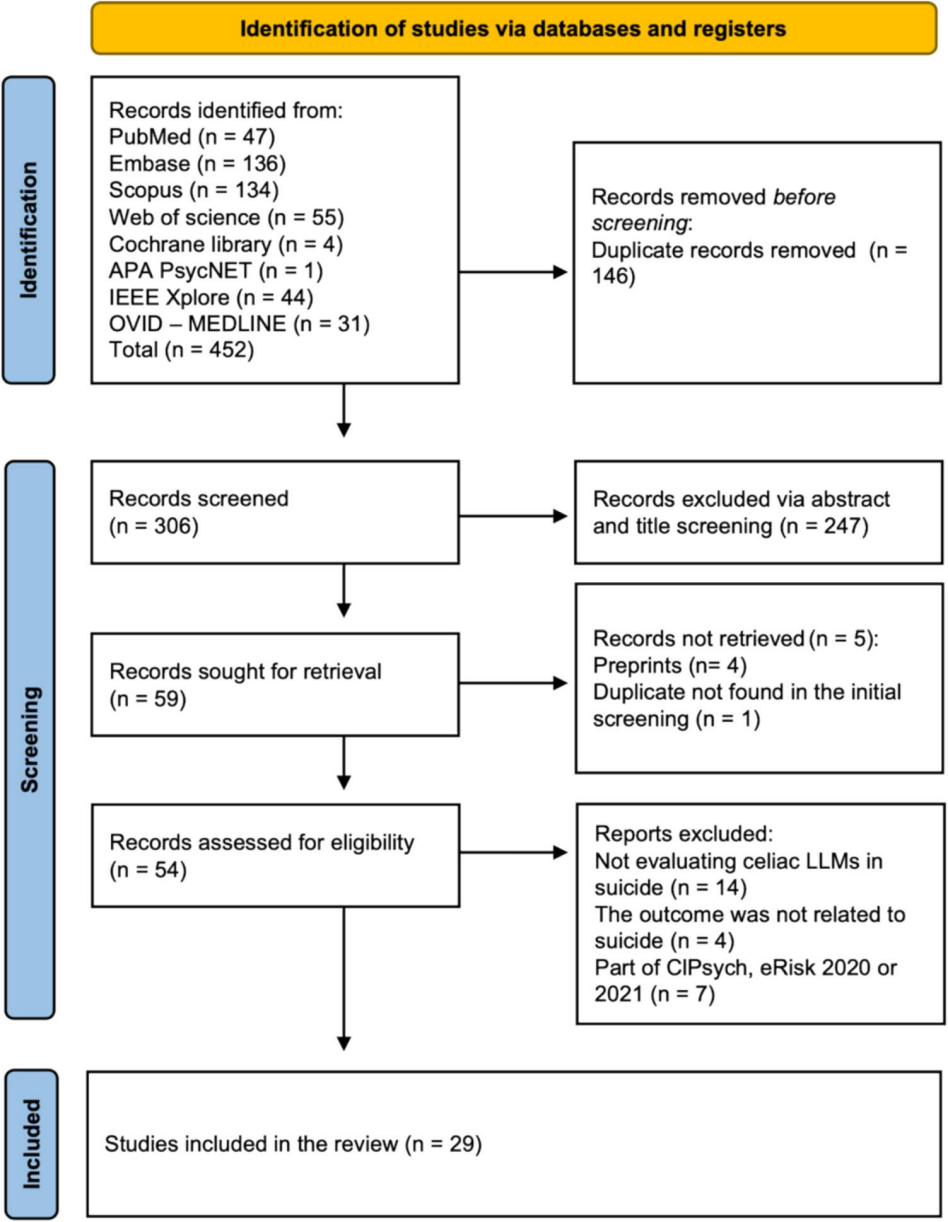
PRISMA flowchart

Our initial search yielded a total of 452 studies. After 146 duplicates were removed, 306 articles remained for title and abstract screening. This screening excluded an additional 247 papers, leaving 59 articles for a full-text review. Five studies were not retrieved because they were either preprints or duplicates that were not initially identified. We also excluded 14 studies that did not evaluate LLM performance in suicide contexts and four studies in which the outcome was not related to suicide. Additionally, we included summary papers from the Workshop on Computational Linguistics and Clinical Psychology [ClPsych 2024] and Early Risk Prediction on the Internet (eRisk) in 2020 and 2021, which provided an overview of results from all participating teams and removed any papers that reported the results of a single participating team. The results of the screening process are shown in the PRISMA flowchart in Fig. 1.

### Risk of Bias

Four studies were assessed using the PROBAST tool, and the remaining 25 studies were assessed using the QUADAS-2 tool. Most studies showed a low risk of bias (ROB) in participant selection (*n* = 20). The index test utilization was also evaluated as having a low ROB in nearly all studies (*n* = 23 out of 25 assessed using QUADAS-2). Similar trends were observed for other evaluation criteria using the two tools. However, many studies exhibited unclear flow and timing (*n* = 10).

Most of the studies showed high applicability concerns (*n* = 22). This was mainly due to participants’ utilization of specific social media sources, often from particular demographics or languages (Tables 2 and 3).

### Overview of the Included Studies

This systematic review included 29 studies published between 2020 and 2024 (12,32–59). These studies utilized various LLMs, such as GPT, Llama, and BERT-Based, and their derivatives. The types of data used in these studies varied, with user-generated content from social media platforms (e.g., Twitter, Reddit), electronic health records (EHR), case reports, and text vignettes the most prominent. Sample sizes ranged from small datasets of 125 Reddit users and medium datasets of 44,506 chat sessions to large datasets of over 1.6 billion labeled tweets (Table 1). Table 1 is organized chronologically by publication year, with studies within each year listed alphabetically by the first author’s last name.

**Table 1:** Summary of included studies

Author	Year	Data Type and Sample Size	Model (Number of parameters)	Task	Results
Haque et al. [65]	2020	Reddit posts (*N* = 3549)	BERT-base (110 M), ALBERT-large (18 M), RoBERTa-large (355 M), XLNet-large (340 M)	Detection	RoBERTa highest performance: Accuracy 95.21%, Recall 98.44%, Precision 92.67%, F1-score 95.47%.
Liu et al. [47]	2020	Weibo posts (*N* = 13,013; annotated: 5994)	CRF, Char-BiLSTM-CRF (BERT embeddings, base 110 M)	Prevention	Best performance: Char-BiLSTM-CRF with BERT embeddings (C_F 0.713, P_F 0.711, E_F 0.688, F_F 0.719).
Losada et al. [43]	2020	Social media posts and comments (Training: *N* = 340; Test: *N* = 423)	LSTM-RNN with BERT (base 110 M)	Detection	Precision up to 0.913, Recall up to 1.0, F1 score up to 0.754, ERDE5 as low as 0.134, Latency-weighted F1 up to 0.658.
Deshpande et al. [32]	2021	Twitter and Reddit posts (*N* = 1.6 billion)m	LSTM-RNN with BERT (base 110 M)	Detection	Training accuracy 96.45%, test accuracy 92.13%; later Reddit data 97% accuracy.
Li et al. [68]	2021	Reddit posts (*N* = 10,000)	BERT-LSTM (BLAM) (base 110 M)	Detection	Accuracy 0.964, Recall 0.971, Precision 0.956, F1-score 0.963.
Parapar et al. [44]	2021	Social media posts and comments (*N* = 1448)	Various models	Detection	Precision 0.532, Recall 0.763, F1 0.627, ERDE5 0.064, ERDE50 0.038, latency-weighted F1 0.622.
Shrestha et al. [48]	2021	Written communications (*N* = 27,329)	RoBERTa-base (125 M)	Prevention	Correctly classified 97% of suicide texts; AUC 0.99, Precision 1.00, Recall 0.97, Specificity 1.00, F1-score 0.99.
Ananthakrishnan et al. [39]	2022	Twitter posts (*N* = 9119)	BERT-based models (e.g., RoBERTa-large, 355 M)	Detection	RoBERTa best performance; accuracy 99.23% (training), 96.35% (validation), 95.39% (testing).
Baghdadi et al. [33]	2022	Arabic tweets (*N* = 14,576)	BERT-base (110 M), Universal Sentence Encoder (USE)	Detection	Best Weighted Sum Metric (WSM) 95.26% (BERT); USE WSM 80.2%.
Castaño et al. [42]	2022	Reddit posts (N = various datasets)	BERT-based models	Detection	Precision up to 91.3%, Latency-weighted F1 0.66, ERDE5 0.134, ERDE50 0.071, F1 Score 0.754.
Metzler et al. [37]	2022	Twitter posts (*N* = 3202)	BERT-base (110 M), XLNet-base (110 M)	Detection	BERT F1-score 0.93 (accurate), 0.74 (less accurate); XLNet similar performance.
Amin et al. [52]	2023	Reddit posts (*N* = 496)	GPT-3 (20 B)	Risk Assessment	ChatGPT Accuracy 92.7%, RoBERTa Accuracy 97.4%, UAR 91.2.
Burkhardt et al. [34]	2023	Social media and clinical texts (*N* = 842)	BERT (base 110 M)	Detection	Improved performance with transfer learning; Best F1 score 0.797; AUROC up to 0.961.
Devika et al. [40]	2023	Reddit posts (N = NR)	BERT (base 110 M), 1D CNN	Detection	Accuracy 99%, loss rate 67%.
Elyoseph et al. [11]	2023	Vignettes (*N* = 379)	GPT-3.5 (20B), GPT-4 (1.8 trillion)	Risk Assessment	GPT-4 Z scores: +0.01 (suicide attempts), + 0.47 (suicidal ideation), + 1.00 (psychache).
Elyoseph et al. [11]	2023	Text vignettes (*N* = 379)	GPT-3.5 (20B), GPT-4 (1.8 trillion)	Risk Assessment	GPT-4 performed comparably to professionals; GPT-3.5 underestimated suicide risk; GPT-4 Z score + 0.01 (suicide attempts).
Ghanadian et al. [51]	2023	Reddit posts (*N* = 21,518)	gpt-3.5-turbo (20B)	Risk Assessment	Zero-Shot Learning: Accuracy 88%, Precision 57%, Recall 100%, F1-score 73%; Few-Shot Learning: Accuracy 81%, Precision 67%, Recall 77%, F1-score 71%.
Izmaylov et al. [54]	2023	Online counseling discussions (*N* = 44,506)	SR-BERT, AlephBERT (base 110 M)	Detection	SR-BERT F2 score 0.76, ROC-AUC 0.92; Ensemble SI-BERT ROC-AUC 0.91.
Kodati et al.	2023	Reddit posts and suicide notes (*N* = 9213)	C-BiGRU-MHA-CNN, L-BiLSTM-MHA-CNN	Prevention	C-BiGRU-MHA-CNN accuracy 98.12%; L-BiLSTM-MHA-CNN accuracy 98.04%.
Murikipudi et al. [49]	2023	EHR (*N* = 12,759)	CMTN	Prevention	Suicide Attempt (SA): Precision 0.97, Recall 0.96, F1-Score 0.96; Suicide Ideation (SI): Precision 0.48, Recall 0.56, F1-Score 0.52.
Spitale et al. [45]	2023	Case reports from Dutch bioethics committees (*N* = 72)	GPT-3.5 (20B)	Prevention	Efficient in classifying case reports and generating plausible cases; 60% plausibility in generated cases.
Wu et al. [41]	2023	Taiwanese social media posts (*N* = 2000)	LSTM, BERT (base 110 M)	Detection	Sensitivity and specificity both 80%.
Zhou et al. [50]	2023	Unstructured narrative reports (*N* = 1462)	FLAN-UL2	Detection	F1-scores over 0.8 for circumstances like sleep problems and sexual violence.
Badian et al. [46]	2024	Facebook images (*N* = 841)	CLIP (400 M), logistic regression	Prevention	AUC 0.720, Cohen’s d 0.82, F1 Score 0.363, NPV 0.934, PPV 0.295, Sensitivity 0.515, Specificity 0.829, ECE 0.041.
Boonyarat et al. [53]	2024	Thai social media tweets (*N* = 2400 annotated, 67,627 analyzed)	Enhanced BERT (base 110 M)	Detection	Suicidal ideation detection F1 score 0.93; Emotion recognition F1 score 0.88.
Chim et al. [12]	2024	Reddit posts (r/SuicideWatch) (*N* = 125)	LLaMA-2 (70 Billion), GPT-3 (20 Billion), BERT	Detection	Highest recall 0.944, highest precision 0.917 (by different models); high consistency scores (e.g., 0.979).
Gorai et al. [64]	2024	Twitter, Reddit posts, and suicide notes (N = NR)	BERT-encoded ensemble CNNs (base 110 M)	Detection	Accuracy up to 99.4%, Precision up to 0.9805, Recall up to 0.9788, F1 score up to 0.9801.
Malhotra et al. [35]	2024	Tweets and Reddit posts (N = various datasets)	BERT derivatives (base 110 M)	Detection	High precision and recall; BERT precision 0.886, recall 0.846 (Twitter); precision 0.967, recall 0.963 (Reddit).
Wang et al. [67]	2024	Reddit posts (*N* = 232,074)	Bi-LSTM, BERT (base 110 M)	Detection	Bi-LSTM accuracy 97%, BERT accuracy 98%.

*AI;* Artificial Intelligence, *AUROC;* Area Under the Receiver Operating Characteristic Curve, *BERT;* Bidirectional Encoder Representations from Transformers, *BiLSTM;* Bidirectional Long Short-Term Memory, *BLAM;* BERT-LSTM with Adversarial and Multi-task Learning, *BoW;* Bag-of-Words, *CCVT;* Caring Contacts Via Text, *CMTN;* Convolutional Multi-Level Transformer Network, *CNN;* Convolutional Neural Network, *CRF;* Conditional Random Field, *ECE;* Expected Calibration Error, *EHR;* Electronic Health Records, *FLAN-UL2;* A large language model approach used in a yes/no question-answer format, *F1;* F1 Score, *LSTM;* Long Short-Term Memory, *GPT;* Generative Pre-trained Transformer, *LSTM-RNN;* Long Short-Term Memory Recurrent Neural Network, *L-BiLSTM-MHA-CNN;* Lexicon-based Bidirectional Long Short-Term Memory with Multi-Head Attention and Convolutional Neural Network, *NLP;* Natural Language Processing, *NPV;* Negative Predictive Value, *PPV;* Positive Predictive Value, *ROC-AUC;* Receiver Operating Characteristic - Area Under the Curve, *SVM;* Support Vector Machine, *USE;* Universal Sentence Encoder, *WSM;* Weighted Sum Metric, *XAI;* Explainable Artificial Intelligence

The tasks these models tested included detection of suicidal thoughts and behaviors, risk assessment, and prevention of suicide attempts. Specifically, 21 papers focused on detection, three on risk assessment, and six on prevention. Most of the studies compared different LLMs or contrasted LLM performance to that of traditional machine learning models, whereas some studies compared LLM performance to that of mental health professionals and manual reviews (Table 1).

#### Analysis of LLM Applications and Performance

**Fig. 2:**
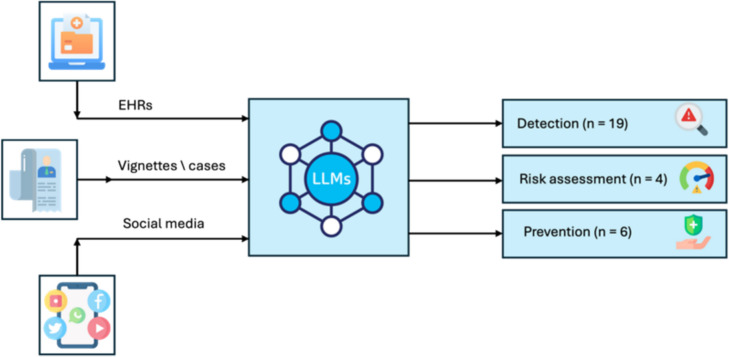
Framework of LLMs tasks and data inputs in suicide detection, assessment, and prevention

We categorized the studies into three main categories according to the task on which the LLM model was evaluated: *detection* of suicidal ideation or behaviors, *risk assessment* of suicidal ideation, and *prevention*, whether directly or by attempting to predict suicide attempts or to detect reasons and circumstances (Fig. 2).

#### Detection of Suicidal Thoughts and Behaviors

Nineteen studies focused on detection of suicidal thoughts and behaviors. Most of this research utilized user-generated content from social media platforms such as Twitter and Reddit, as well as other sources such as EHRs and text vignettes. Several models were employed across studies, with BERT and its variations the most prominent.

For example, Deshpande et al. [32] used an LSTM-RNN with BERT encoding to achieve a test accuracy of 92.13%, which increased to 97% with additional Reddit data [32]. Baghdadi et al. [33] used BERT and the Universal Sentence Encoder to classify Arabic tweets, achieving a best Weighted Sum Metric (WSM) of 95.26%, demonstrating high effectiveness in detecting suicidal thoughts [33].

Transfer learning also emerged as a notable strategy for enhancing model performance. Burkhardt et al. [34]applied a multi-stage transfer learning strategy using BERT, combining data from Reddit and clinical text messages from a Caring Contacts Via Text (CCVT) clinical trial [34]. This approach significantly improved the performance metrics over baseline models, achieving an F1 score of 0.797 and an AUROC of up to 0.961 [34].

Malhotra et al. [35] employed six pre-trained Transformer-based LLMs, including BERT and RoBERTa, to detect depressive and suicidal behaviors on social networks [35]. These models, combined with Explainable AI (XAI) techniques such as SHapley Additive exPlanations (SHAP) and Local Interpretable Model-agnostic Explanations (LIME), demonstrated high precision and recall across different datasets [36]. BERT, for instance, achieved a precision of 0.886 and a recall of 0.846 on Twitter data and a precision of 0.967 and a recall of 0.963 on Reddit data [36].

Performance varied across the datasets and tasks. For example, Metzler et al. [37] demonstrated that BERT and XLNet effectively differentiated harmful from protective suicide-related content on Twitter, achieving high accuracy and F1-scores [37]. On the binary classification task, BERT achieved an F1-score of 0.93 for classifications we define as accurate, indicating correct identification of suicidal intent in user-generated content [37]. In contrast, ‘less accurate classifications,’ with an F1-score of 0.74, refer to instances where the model struggled to distinguish ambiguous expressions that may or may not indicate suicidal ideation [38] (Tables 2 and 3).

**Table 2:** Assessment of diagnostic studies using Quality Assessment of Diagnostic Accuracy Studies-2 (QUADAS-2)

	Risk of Bias			Applicability concerns			
Author	Patient selection	Index test	Reference standard	Flow and timing	Patient selection	Index test	Reference standard
Spitale et al. [45]	High	Low	Low	Low	High	Low	Low
Elyoseph et al. [11]	High	Low	Low	Unclear	High	Low	Low
Burkhardt et al. [34]	Low	Low	Low	Unclear	High	Low	Low
Deshpande et al. [32]	Low	Low	Low	Low	Low	Low	Low
Zhou et al. [50]	Low	Low	Low	High	Low	Low	High
Malhotra et al. [35]	Low	High	Low	Low	Low	Low	Low
Metzler et al. [37]	Low	Low	Low	Low	High	Low	Low
Baghdadi et al. [33]	Low	Low	High	High	High	Low	High
Ananthakrishnan et al. [39]	Low	Low	High	Low	High	Low	High
Devika et al. [40]	High	Low	High	Unclear	High	Low	High
Gorai et al. [64]	High	Low	High	Unclear	High	Low	High
Chim et al. [12]	High	Low	High	Unclear	High	Low	High
Wu et al. [41]	Low	Low	Low	Low	Low	Low	Low
Wang et al. [67]	Low	Low	Low	Low	High	Low	Low
Izmaylov et al. [54]	Low	Low	Low	Unclear	High	Low	Low
Boonyarat et al. [53]	Low	Low	Low	Low	High	Low	Low
Ghanadian et al. [51]	Low	Low	High	High	High	Low	High
Castaño et al. [42]	Low	Low	Low	Unclear	High	Low	High
Losada et al. [43]	High	High	High	Unclear	High	High	High
Li et al. [68]	Low	Low	Low	Low	Low	Low	Low
Parapar et al. [44]	Low	Low	Low	Unclear	High	Low	Low
Haque et al. [65]	Low	Low	Low	Low	High	Low	Low
Murikipudi et al. [49]	Low	Low	Low	Low	Low	Low	Low
Soudi et al. [66]	High	Low	High	Unclear	High	Low	High
Amin et al. [52]	Low	Low	Low	Low	High	Low	Low

**Table 3:** Assessment of predictive studies using the prediction model risk of Bias Assessment Tool (PROBAST)

	Risk of bias				Applicability		
Author	Participants	Predictors	Outcome	Analysis	Participants	Predictors	Outcome
Elyoseph et al. [11]	High	Low	Low	Low	High	Low	Low
Badian et al. [46]	High	Low	Low	Low	High	Low	Low
Liu et al. [47]	Low	Low	Low	Low	High	Low	Low
Shrestha et al. [48]	Low	Low	Low	Low	Low	Low	Low

Other studies also emphasized the effectiveness of BERT-based models. Ananthakrishnan et al. [39] compared five BERT-based models in detecting suicidal intentions from tweets and found that RoBERTa achieved the best performance, with accuracy rates of 99.23% in training, 96.35% in validation, and 95.39% in testing [39]. Devika et al. [40] combined BERT with a 1D Convolutional Neural Network (1D CNN) to identify depression and suicidal thoughts in social media posts and reported an accuracy of 99% [40].

Challenges in ensuring generalizability and maintaining accuracy in real-world scenarios have been reported across studies. For example, a study by Wu et al. [41] on Taiwanese social media platforms highlighted the limitations of data from specific platforms and the need for broader applicability [41]. Castaño et al. [42] emphasized the importance of reliable user-generated content, noting potential biases in subreddit data [42]. The eRisk shared tasks [43, 44] demonstrated variability in performance, with models achieving precision of up to 0.913 and F1 scores of up to 0.754, but also highlighted the need for further research to improve prediction accuracy [43, 44].

#### Suicide Prevention

Six studies focused on suicide prevention, either directly or indirectly, by researching reasons for suicide, predicting suicidal ideation, or analyzing circumstances.

Spitale et al. [45] explored the use of GPT-3.5 to classify and generate case reports on assisted suicide from the Dutch Bioethics Committees’ database. The model demonstrated efficiency in categorizing case reports and generated fictional cases with a plausibility rate of 60%. This study highlighted AI’s potential in generating practice cases that can help understand and prevent suicidal behaviors [45].

Badian et al. [46] focused on predicting high suicide risk based on images uploaded to Facebook. They used a hybrid model that combined contrastive language image pre-training (CLIP) with logistic regression, achieving an AUC of 0.720 [46]. This study demonstrates that publicly available images on social media can be utilized effectively to predict validated suicide risk [46]. However, the study was limited by its reliance on self-reported data and its sole focus on image data.

Liu et al. [47] developed a model for extracting suicidal ideation causes (SIC) from social texts on Weibo. They compared several models, including CRF and Char-BiLSTM-CRF, with different word embeddings, such as Word2vec, ELMo, and BERT. The Char-BiLSTM-CRF model with BERT embeddings achieved the highest performance, showing a significant advantage over traditional models.

Shrestha et al. [48] investigated identification of suicidal risk in written communications, including suicide notes, forum posts, social media posts, and blog entries. They used RoBERTa and compared it to traditional machine-learning models, such as SVM. RoBERTa correctly classified 97% of the suicide texts, outperforming SVM and linguistic marker models [48].

Murikipudi et al. [49] applied a Convolutional Multi-Level Transformer Network (CMTN) to identify suicidal behaviors using EHR data from 12,759 inpatient hospital stays. The CMTN model exhibited high performance in detecting suicidal attempts (SA), with a precision rate of 0.97 and a recall rate of 0.96. For suicidal ideation (SI), the model demonstrated a precision rate of 0.48 and a recall rate of 0.56. Nevertheless, data imbalance and the specificity of the extraction algorithms pose challenges [49].

Zhou et al. [50] used the FLAN-UL2 model to identify the rare circumstances that preceded female firearm suicides from narrative reports. The model outperformed traditional SVM approaches, achieving F1-scores over 0.8 for specific circumstances, such as sleep problems and sexual violence [50].

#### Risk Assessment

Elyoseph and Levkovich [38] used text vignettes to compare GPT-3.5 with mental health professionals [38]. Their study found that GPT-3.5 consistently underestimated suicide risk compared to mental health professionals, with assessments in the 5th percentile for risk ratings (*p* < 0.001). This result highlights the model’s limitations in terms of accurately assessing severe scenarios. Another study Levkovich and Elyoseph [48] compared the performance of GPT-3.5 and GPT-4 to that of mental health professionals in assessing suicide risk. The researchers used vignettes depicting hypothetical patients with varying levels of perceived burdensomeness and thwarted belongingness based on the interpersonal theory of suicide. The study found that GPT-4’s performance was comparable to that of mental health professionals, with an average Z score of + 0.01 in assessing risk of suicide attempts. However, GPT-4 tended to overestimate psychache and suicidal ideation, with Z-scores of + 1.00 and + 0.47, respectively. GPT-3.5 generally underestimated suicide risk compared to professionals [48].

Ghanadian et al. [51] evaluated GPT (GPT-3.5-turbo) alongside ALBERT and DistilBERT in assessing the level of suicidality in social media posts from subreddits such as SuicideWatch and Depression. They used Zero-Shot and Few-Shot Learning techniques. In Zero-Shot Learning, GPT achieved an accuracy of 88%, precision of 57%, recall of 100%, and F1-score of 73%. In Few-Shot Learning, the model attained an accuracy of 81%, precision of 67%, recall of 77%, and F1-score of 71%. Nevertheless, the fine-tuned ALBERT model outperformed GPT with an F1-score of 86.9%, whereas DistilBERT had an F1-score of 74.5% [51].

Amin et al. [52] examined the performance of GPT (fine-tuned with Reinforcement Learning from Human Feedback) in assessing suicide risk from Reddit posts in subreddits such as SuicideWatch, Depression, and Teenagers. This study compared GPT performance to that of RoBERTa-base, Word2Vec, and Bag-of-Words (BoW). GPT achieved an accuracy rate of 92.7%, but RoBERTa outperformed GPT, with an accuracy rate of 97.4% and an Unweighted Average Recall of 91.2. The study found that although GPT demonstrated decent generalist performance across affective computing tasks, it did not outperform specialized models such as RoBERTa [52].

#### Validation

Sixteen studies reported using classical validation techniques, such as n-fold cross-validation or separate training and test data, while fourteen did not specify these approaches in detail.

Among the Sixteen studies that applied validation techniques, cross-validation was commonly used. For example, Spitale et al. [45] utilized 10-fold cross-validation with L2 regularization to prevent overfitting, while Burkhardt et al. [34] applied 5-fold cross-validation on clinical data, ensuring model reliability by reporting median scores across different random splits. Other studies, such as those by Deshpande and Warren [32], used an 80:20 train-test split, allowing BERT models to be evaluated on separate test sets, while Badian et al. [46] and Boonyarat et al. [53] followed similar approaches with varying train-test ratios, repeating random splits to address potential imbalances.

The remaining fourteen studies either lacked independent validation or reported alternative validation techniques without traditional data splits. Some, like Elyoseph et al. [38], used vignettes for model assessment without employing test sets or cross-validation, while Levkovich and Elyoseph [48] validated against mental health norms but without data partitioning. Others, such as Malhotra and Jindal [35], relied on few-shot learning and explainability tools like SHAP and LIME, focusing on model interpretability rather than performance validation. Wu et al. [41] combined NLP-based predictions with manual professional validation, whereas Izmaylov et al. [54] implemented initial validation via pre-training steps but did not include explicit cross-validation or hold-out test sets. Finally, Losada et al. [43] and Parapar et al. [44] applied sequential real-time monitoring techniques in eRisk studies, using metrics like ERDE and latency-weighted F1 but without traditional validation approaches.

## Discussion

This review aimed to investigate the application of LLM tools in diverse facets of suicide prevention. The 29 reviewed studies were systematically categorized based on the specific tasks for which the LLM models were evaluated, yielding three primary categories: detection of suicidal ideation or behaviors, risk assessment of suicidal ideation, and prevention, either directly or through prediction of suicide and suicide attempts.

In the current review, 19 of the 29 studies focused on the identification of suicidal thoughts and behaviors. Much of the research leveraged user-generated content from social media platforms, such as Twitter and Reddit, in addition to other sources, such as electronic health records (EHRs) and text vignettes. Several models demonstrated high encoding capabilities that achieved a test accuracy ranging from 92 to 97% in detecting suicidal thoughts [32–34]. Yet discrepancies were observed across research methodologies and systems. For instance, one study found that the performance of GPT-3.5 was generally underestimated, as reported by Elyoseph et al. [38]. Our review aligns with those findings, highlighting the need for further evaluation. Likewise, another recent review explored the detection of suicidal ideation and behavior using audiovisual features, such as voice acoustics and visual cues [24]. While these non-verbal indicators are promising for assessing mental disorders, the existing literature is limited to a few small studies, as noted by Dhelim et al. [24]. These gaps underscore the need for more comprehensive research in this area [24]. A review of natural language processing approaches for suicide risk detection revealed that despite the varied methodologies and small sample sizes, these techniques consistently outperformed human raters in accurately identifying suicide risk [36].

Six of the 29 studies in this review focused on suicide prevention. Relatively few studies have addressed this topic, particularly those offering practice and training to professionals. According to the evidence from the included studies, the risk of suicide can be effectively predicted by analyzing images or texts from social networks [33, 52]. These models demonstrated moderate efficiency (approximately 60%) [33, 52]. Moreover, higher efficiency was observed in more complex studies that use social media texts, such as Liu et al. [47]. The highest accuracy (97%) was reported in Shrestha et al. [48], which incorporated a model analysis of social networks, suicide notes, and other sources to predict suicide [58]. Public health suicide prevention programs have been shown to reduce suicide rates significantly. These include gatekeeper programs and specialized initiatives in organizations, the military, police, schools, and more, with some studies reporting an effectiveness rate of 30–57% [55, 56]. While effectiveness varies across different populations, police officers demonstrated a greater ability to prevent suicide, whereas educators showed less ability [63].

Integrating LLMs into suicide prevention appears promising, demonstrating good results despite the small sample sizes in the studies. This association between LLMs’ predictive capabilities and the success of public health programs highlights the potential of combining advanced AI models with traditional prevention strategies to save lives.

## Limitations

This study has several limitations. First, several of the examined studies relied on synthetic data, which may not capture the complexities of the real world and may require human verification [64]. Moreover, some studies used single, female-focused vignettes, thus limiting the generalizability of the results, and some only tested one AI model against a sample of mental health professionals. Clinical datasets are often small and specific and social media data may have potential biases and diverse characteristics. Additionally, some models may face challenges with language variability in tweets and biases owing to non-representative samples. Furthermore, many models lack the ability to explain their predictions, an ability that is crucial for helping counselors.

Four out of the 29 articles focused on suicide risk assessment. These studies highlight some limitations of LLMs, particularly in vignette studies. For instance, GPT models were found to produce inconsistent assessments or overestimations ]38[. Better results were observed in the studies by Ghanadian et al. and Amin et al. [51, 52]. Amin et al. reported that while RoBERTa outperformed GPT, GPT still achieved an accuracy of 92.7% [57]. Similarly, Ghanadian et al. found that a hyperparameter-optimized GPT model (e.g., temperature) showed potential for suicide risk assessment in Zero-Shot Learning, with an accuracy of 88% and an F1-score of 73% ]39[.

The research emphasizes the intricacy of evaluating an individual’s risk of suicide. The evidence indicates that incorporating LLMs can significantly improve clinical decision-making in suicide risk assessments among professionals [12, 35, 47, 49–51]. Furthermore, LLMs can enhance training and clinical procedures for mental health and medical professionals [51]. Their accessibility may also contribute to reducing the stigma surrounding mental health and encourage individuals to seek help [16].

Nevertheless, integrating LLMs into suicide risk detection presents several challenges. The reliability of LLM predictions depends heavily on the quality and demographic inclusivity of the training data [65]. Data biases or insufficient demographic representation may lead to inaccurate predictions or exacerbate existing health disparities [65]. Moreover, the opacity of ChatGPT’s algorithms can obscure the reasoning behind its predictions, which may hinder trust and acceptance among users [66]. The use of LLMs for suicide risk assessment also raises ethical concerns. Ensuring data privacy and security is crucial, particularly given the sensitive nature of mental health information. Users must be fully informed about how their data is used and protected. ChatGPT should not be used to supplant human clinical judgment but rather to complement it by assisting professionals in making more informed decisions.

Implementing LLMs in suicide prevention raises several ethical concerns that necessitate careful consideration [11]. Privacy and confidentiality are paramount, as LLMs process highly sensitive mental health data. Ensuring data security and adherence to privacy laws is essential to protect users’ personal information from misuse or breaches [57]​. Another significant issue is algorithmic bias; LLMs trained on datasets lacking demographic diversity may inadvertently reinforce cultural, gender, or socioeconomic biases, potentially leading to inequitable mental health assessments [19, 58, 59]​. This is particularly problematic in suicide prevention, where accurate assessments are critical. The ‘black box’ nature of LLMs presents another ethical challenge, as the opacity of AI decision-making can make it difficult for clinicians to trust these models or understand their outputs. This lack of interpretability could limit their acceptance and application in clinical settings [60]​. Furthermore, while AI tools can support mental health professionals, there is a risk of over-reliance on LLMs, potentially shifting focus away from human-centered care. Experts caution that LLMs should serve as complementary tools rather than replacements for clinical expertise, which remains essential for understanding the complexities of mental health [17]. Addressing these ethical issues will require interdisciplinary collaboration and continuous research to establish guidelines that prioritize both efficacy and ethical integrity.

One of the most promising aspects of LLMs in mental health and suicide prevention is their ability to be rapidly deployed across multiple languages, including those traditionally underrepresented in mental health resources [12, 17, 54]. This multilingual capability is essential for reducing language barriers and making life-saving interventions accessible to a broader population [61]. Advanced models like GPT-4 and mT5 have demonstrated high accuracy in cross-linguistic translation and nuanced contextual understanding, making them ideal for adapting suicide prevention tools to low-resource languages [38, 41, 62]. Such advancements in language inclusivity are crucial in mental health settings, as they enhance crisis response across diverse linguistic and cultural contexts [58]. In parallel, emerging research on GenAI-based simulators has shown promise in strengthening suicide risk assessment skills among mental health professionals. Professionals’ self-efficacy in assessing suicidality and their willingness to treat at-risk patients improved after engaging with these simulators, highlighting their potential as training tools in suicide prevention [63].

The field of LLMs is progressing at an unprecedented pace, with newer models achieving remarkable improvements in natural language understanding and contextual accuracy. Models like GPT-4 and others demonstrate significant advancements in processing and interpreting complex linguistic inputs, enhancing their utility in sensitive applications such as mental health [41, 54]. As larger and more sophisticated models continue to be developed, the precision and reliability of tools for suicide detection and risk assessment are expected to improve. For example, recent studies with advanced models have shown significant improvements in nuanced understanding and accuracy in complex tasks, suggesting that the limitations observed in current studies may soon be mitigated [12]. This rapid evolution positions LLMs to become an even more effective component of mental health strategies, enhancing diagnostic accuracy and therapeutic support [17].

## Conclusion

In this systematic literature review, we aimed to characterize the existing body of research on large language models in the context of suicide recognition and prevention. We identified 29 peer-reviewed publications published from 2020 to 2024 that utilized LLMs. We provided a comprehensive overview and categorized the research into three main areas: detection of suicidal ideation or behaviors, risk assessment of suicidal ideation, and prevention, either directly or by predicting suicides and attempts. Most of the studies demonstrated high effectiveness relative to the detection and prediction abilities of mental health professionals.

The potential of LLMs cannot be disregarded. Indeed, most publications demonstrate a significant advantage for LLMs, especially when compared to existing literature in the field. Nevertheless, the research methods used in these studies vary considerably. Moreover, ethical issues regarding the use of AI for mental health must be highlighted. Follow-up studies are needed to understand how AI can assist professionals in preventing, identifying, and intervening in suicide cases.

## Supplementary Information

Below is the link to the electronic supplementary material.

ESM 1 (DOCX 17.6 KB)

## Data Availability

No datasets were generated or analysed during the current study.

## Abbreviations

LLM: Large Language Models
GPT: Generative Pretrained Transformer
BERT: Bidirectional Encoder Representations from Transformers
XAI: Explainable AI
EHR: Electronic Health Records
AUC: Area Under the Curve
