# Supplementary material for: Evaluating of BERT-based and Large Language Mod for Suicide Detection, Prevention, and Risk Assessment: A Systematic Review
Source: J Med Syst. 2024 Dec 30;48(1):113. doi: 10.1007/s10916-024-02134-3 (PMC11685247; doi:10.1007/s10916-024-02134-3)
Supplement: Supplementary file 1 — (DOCX 17.6 KB) [file 10916_2024_2134_MOESM1_ESM.docx]

**Supplementary materials**

**Evaluating the Efficacy of Large Language Models for Suicide Detection, Prevention, and Risk Assessment : A Systematic Review**

**Specific Booleans used to search each database:**

**PubMed**

(("large language model" OR "LLM" OR "LLMs" OR "GPT" OR "ChatGPT" OR "GPT-3" OR "GPT-3.5" OR "GPT-4" OR "Generative Pre-trained Transformer" OR "BERT" OR "Bidirectional Encoder Representations from Transformers" OR "Transformer models" OR "RoBERTa" OR "XLNet" OR "AI language model") AND (suicide OR "suicidal ideation" OR "self-harm" OR "suicide attempt" OR "suicidal thoughts"))

**Embase**

('large language model'/exp OR 'large language model' OR 'llm' OR 'llms' OR 'gpt'/exp OR 'gpt' OR 'chatgpt'/exp OR 'chatgpt' OR 'gpt-3' OR 'gpt-3.5' OR 'gpt-4' OR 'generative pre-trained transformer'/exp OR 'generative pre-trained transformer' OR 'bert' OR 'bidirectional encoder representations from transformers'/exp OR 'bidirectional encoder representations from transformers' OR 'transformer models' OR 'roberta' OR 'xlnet' OR 'ai language model') AND ('suicide'/exp OR suicide OR 'suicidal ideation'/exp OR 'suicidal ideation' OR 'self-harm'/exp OR 'self-harm' OR 'suicide attempt'/exp OR 'suicide attempt' OR 'suicidal thoughts'/exp OR 'suicidal thoughts')

AND

(2018:py OR 2019:py OR 2020:py OR 2021:py OR 2022:py OR 2023:py OR 2024:py)

AND

'article'/it

AND

[embase]/lim NOT ([embase]/lim AND [medline]/lim)

**OVID – MEDLINE**

('large language model'/exp OR 'large language model' OR 'llm' OR 'llms' OR 'gpt'/exp OR 'gpt' OR 'chatgpt'/exp OR 'chatgpt' OR 'gpt-3' OR 'gpt-3.5' OR 'gpt-4' OR 'generative pre-trained transformer'/exp OR 'generative pre-trained transformer' OR 'bert' OR 'bidirectional encoder representations from transformers'/exp OR 'bidirectional encoder representations from transformers' OR 'transformer models' OR 'roberta' OR 't5' OR 'xlnet' OR 'ai language model') AND ('suicide'/exp OR suicide OR 'suicidal ideation'/exp OR 'suicidal ideation' OR 'self-harm'/exp OR 'self-harm' OR 'suicide attempt'/exp OR 'suicide attempt' OR 'suicidal thoughts'/exp OR 'suicidal thoughts')

**Scopus**

( TITLE-ABS-KEY ( "large language model" OR "LLM" OR "LLMs" OR "GPT" OR "ChatGPT" OR "GPT-3" OR "GPT-3.5" OR "GPT-4" OR "Generative Pre-trained Transformer" OR "BERT" OR "Bidirectional Encoder Representations from Transformers" OR "Transformer models" OR "RoBERTa" OR "XLNet" OR "AI language model" ) AND TITLE-ABS-KEY ( suicide OR "suicidal ideation" OR "self-harm" OR "suicide attempt" OR "suicidal thoughts" ) ) AND PUBYEAR > 2017 AND PUBYEAR < 2025 AND ( EXCLUDE ( DOCTYPE , "cr" ) OR EXCLUDE ( DOCTYPE , "re" ) OR EXCLUDE ( DOCTYPE , "ed" ) OR EXCLUDE ( DOCTYPE , "no" ) OR EXCLUDE ( DOCTYPE , "er" ) OR EXCLUDE ( DOCTYPE , "bk" ) ) AND ( LIMIT-TO ( LANGUAGE , "English" ) )

**Web of science**

(TS=("large language model" OR "LLM" OR "LLMs" OR "GPT" OR "ChatGPT" OR "GPT-3" OR "GPT-3.5" OR "GPT-4" OR "Generative Pre-trained Transformer" OR "BERT" OR "Bidirectional Encoder Representations from Transformers" OR "Transformer models" OR "RoBERTa" OR "XLNet" OR "AI language model") AND TS=(suicide OR "suicidal ideation" OR "self-harm" OR "suicide attempt" OR "suicidal thoughts"))

**APA PsycNET**

((AB "large language model" OR AB "LLM" OR AB "LLMs" OR AB "GPT" OR AB "ChatGPT" OR AB "GPT-3" OR AB "GPT-3.5" OR AB "GPT-4" OR AB "Generative Pre-trained Transformer" OR AB "BERT" OR AB "Bidirectional Encoder Representations from Transformers" OR AB "Transformer models" OR AB "RoBERTa" OR AB "T5" OR AB "XLNet" OR AB "AI language model") AND (AB suicide OR AB "suicidal ideation" OR AB "self-harm" OR AB "suicide attempt" OR AB "suicidal thoughts"))

**Cochrane library**

(("large language model" OR "LLM" OR "LLMs" OR "GPT" OR "ChatGPT" OR "GPT-3" OR "GPT-3.5" OR "GPT-4" OR "Generative Pre-trained Transformer" OR "BERT" OR "Bidirectional Encoder Representations from Transformers" OR "Transformer models" OR "RoBERTa" OR "XLNet" OR "AI language model") AND (suicide OR "suicide" OR "suicidal ideation" OR "self-harm" OR "suicide attempt" OR "suicidal thoughts"))

**IEEE Xplore**

(("large language model" OR "LLM" OR "LLMs" OR "GPT" OR "ChatGPT" OR "GPT-3" OR "GPT-3.5" OR "GPT-4" OR "Generative Pre-trained Transformer" OR "BERT" OR "Bidirectional Encoder Representations from Transformers" OR "Transformer models" OR "RoBERTa" OR "XLNet" OR "AI language model") AND (suicide OR "suicidal ideation" OR "self-harm" OR "suicide attempt" OR "suicidal thoughts"))
